# Infraclavicular nerve block reduces postoperative pain after distal radial fracture fixation: a randomized controlled trial

**DOI:** 10.1186/s12871-020-01044-4

**Published:** 2020-05-28

**Authors:** Stanley S. Wong, Wing S. Chan, Christian Fang, Chi W. Chan, Tak W. Lau, Frankie Leung, Chi W. Cheung

**Affiliations:** 1 Laboratory and Clinical Research Institute for Pain, Department of Anaesthesiology, Li Ka Shing Faculty of Medicine, The University of Hong Kong, Queen Mary Hospital, Room 424, Block K, 102, Pokfulam Road, Hong Kong SAR, China; 2 Department of Orthopaedics and Traumatology, Li Ka Shing Faculty of Medicine, The University of Hong Kong, Hong Kong SAR, China; 3 Department of Anaesthesiology, Queen Mary Hospital, Hong Kong SAR, China; 4 Department of Orthopaedics and Traumatology, Queen Mary Hospital, Hong Kong SAR, China

**Keywords:** General anesthesia, Regional anesthesia, Distal radial fracture fixation, Postoperative pain, Infraclavicular nerve block

## Abstract

**Background:**

It is unclear whether regional anesthesia with infraclavicular nerve block or general anesthesia provides better postoperative analgesia after distal radial fracture fixation, especially when combined with regular postoperative analgesic medications. The aim of this study was to compare the postoperative analgesic effects of regional versus general anesthesia.

**Methods:**

In this prospective, observer blinded, randomized controlled trial, 52 patients undergoing distal radial fracture fixation received either general anesthesia (*n* = 26) or regional anesthesia (infraclavicular nerve block, n = 26). Numerical rating scale pain scores, analgesic consumption, patient satisfaction, adverse effects, upper limb functional scores (Patient-Rated Wrist Evaluation, QuickDASH), health related quality of life (SF12v2), and psychological status were evaluated after surgery.

**Result:**

Regional anesthesia was associated with significantly lower pain scores both at rest and with movement on arrival to the post-anesthetic care unit; and at 1, 2, 24 and 48 h after surgery (*p* ≤ 0.001 at rest and with movement). Morphine consumption in the post-anesthetic care unit was significantly lower in the regional anesthesia group (p<0.001). There were no differences in oral analgesic consumption. Regional anesthesia was associated with lower incidences of nausea (*p* = 0.004), and vomiting (*p* = 0.050). Patient satisfaction was higher in the regional anesthesia group (*p* = 0.003). There were no long-term differences in pain scores and other patient outcomes.

**Conclusion:**

Regional anesthesia with ultrasound guided infraclavicular nerve block was associated with better acute pain relief after distal radial fracture fixation, and may be preferred over general anesthesia.

**Trial registration:**

Before subject enrollment, the study was registered at ClinicalTrials.gov (NCT03048214) on 9th February 2017.

## Background

Distal radial fracture fixation is a commonly performed orthopedic surgery. It is usually associated with moderate pain, but can sometimes also result in severe postoperative pain. Poor postoperative pain control would interfere with rehabilitation, delay recovery, and adversely affect outcomes [1, 2]. Distal radial fracture fixation is performed either under general anesthesia (GA) or regional anesthesia (RA) using a brachial plexus block. The choice of anesthetic technique may affect postoperative pain control.

Regional anesthetic nerve blocks have been shown to reduce acute postoperative pain, opioid consumption, improve patient satisfaction, and shorten postoperative stay [3–6]. However, 2 randomized controlled trials found no overall difference between RA and GA for analgesia after distal radial fracture fixation [7, 8]. Instead, RA was associated with rebound pain and worse pain scores compared with GA at 24 h after surgery. However, standardized regular postoperative analgesics were not given to prevent rebound pain in these clinical trials. Regular analgesic medication is recommended for optimal postoperative pain control and prevention of rebound pain [1, 2, 9–11]. The use of regular postoperative analgesic medication could positively affect the analgesic effect of RA and reduce rebound pain after distal radial fracture fixation. To the best of our knowledge, this has not been studied.

We performed a randomized controlled trial to compare the acute postoperative analgesic effect of RA with ultrasound guided infraclavicular nerve block versus GA plus local anesthetic would infiltration for distal radial fracture fixation. This was conducted with the use of regular postoperative analgesic medication. We also looked at longer-term secondary outcomes including chronic pain, upper limb functional scores, health related quality of life, and psychological well-being at 3 and 6 months after surgery. We hypothesized that RA together with regular postoperative analgesic medication would improve acute postoperative pain control after distal radial fracture fixation.

## Methods

This study was conducted in a tertiary university hospital in Hong Kong, China. It was approved by the local university’s Institutional Review Board (UW 16–005), and registered at clinicaltrial.gov prior to patient recruitment on 9th February 2017 (NCT03048214). Written consent was obtained from all patients participating in the trial.

This was a prospective, observer blinded, randomized controlled trial. Patients aged between 18 and 80 years old with an American Society of Anesthesiologist (ASA) physical status of I-III scheduled for distal radial fracture fixation (open reduction and internal fixation) were eligible. Exclusion criteria included surgery involving more than fixation of one affected arm; allergy to analgesic drugs including opioids, non-steroidal anti-inflammatory drugs (NSAIDs), local anesthetic drugs, and paracetamol; presence of chronic pain condition; chronic opioid user; alcohol or substance abuse; impaired renal function (defined as serum creatinine level over 120 μmol/L; impaired liver function (defined as plasma bilirubin over 34 μmol/L, international normalized ratio (INR) ≥1.7, alanine aminotransferase (ALT) and aspartate aminotransferase (AST) over 100 U/L; pre-existing neurological or muscular disorders; psychiatric illness; impaired mental state; non-ambulatory; pregnancy; local infection; coagulopathy or on anticoagulants (not including aspirin); or patient refusal.

Eligible patients were randomly assigned to receive either GA plus local anesthetic wound infiltration (GA group) or RA with infraclavicular nerve block (RA group). Patients were randomized using a computer-generated random sequence. A statistician unaware of the nature of the clinical study prepared the sequence. Allocation was concealed in opaque envelopes and opened at the time of intervention by the attending anesthetist. Patients were aware of the type of anesthesia they were receiving. An investigator who was blinded from patient allocation collected the data from the patients. Patients were informed upon enrollment into the study not to tell the investigator collecting data which group they belonged to. The anesthetist who performed the anesthetic was aware of patient allocation, but was not involved in data collection.

Sedative premedication was not prescribed. On arrival to the operating theatre, a 20- or 22-gauge intravenous cannula was inserted. Standard monitoring with pulse oximeter, non-invasive blood pressure, and three lead electrocardiogram (ECG) were applied before induction. Non-invasive blood pressure was measured at least every 5 min throughout the operation. Distal radial fracture fixation was performed in the hospital trauma list, which operated from 8.30 am to 16:30 pm on every weekday.

### General anesthesia group (GA)

Patients in the GA group were induced with intravenous propofol 1.5-3 mg/kg, fentanyl 0.25-2mcg/kg and atracurium 0.5 mg/kg. Patients were either intubated with an endotracheal tube or given a laryngeal mask airway as determined by the attending anesthetist. Additional doses of muscle relaxants were given as needed. GA was maintained with sevoflurane, air and oxygen (oxygen and air titrated to give a fraction of inspired oxygen (FiO_2_) of between 35 and 50%). Sevoflurane was titrated to 0.7–1.5 minimum alveolar concentration (MAC), and nitrous oxide was not used.

Intravenous morphine sulphate at a dose of 0.05–0.1 mg/kg was given immediately prior to surgical incision. Additional boluses of morphine sulphate at 0.025–0.05 mg/kg could be given at the discretion of the attending anesthetist. Around thirty minutes before end of surgery, intravenous paracetamol at a dose of 1000 mg (15 mg/kg for patients under 50 kg) was given. Ondansetron 4 mg was given intravenously 30 min before the end of surgery. Sevoflurane was switched off after the inner layers of wound were closed. Local wound infiltration with 2 mg/kg of 0.5% levobupivacaine was given by the surgeon during wound closure. Reversal of muscle relaxation was achieved with neostigmine 50mcg/kg and atropine 20mcg/kg given intravenously. Subsequently, the patient was transferred to the post-anesthetic care unit (PACU) for 30 min to 1 h.

### Regional anesthesia group (RA)

Infraclavicular nerve blocks were performed under ultrasound guidance using a 22-gauge 50 mm or 100 mm Pajunk® needle for patients in the RA group. The decision whether to use a nerve stimulator was left to the attending anesthetist. Local anesthetic solution was made up of 10 ml of 2% lignocaine with 1:200,000 adrenaline plus 10 ml of 0.75% ropivacaine, made up to a total of 20 ml of local anesthetic solution. Fifteen to twenty milliliters of local anesthetic solution were deposited around the lateral, posterior, and medial cord of the brachial plexus under ultrasound guidance. All regional blocks were performed by a skilled specialist anesthetist or resident fellow under the direct supervision of a specialist anesthetist competent in performing ultrasound guided infraclavicular nerve blocks. Any immediate complications including paresthesia and vascular puncture were noted.

Patients were sedated with propofol infusion during surgery by using the Marsh effect site model to keep the effect site concentration between 0.5–1.5mcg/ml. Propofol level was titrated to put the patients under light sleep where they could be easily aroused with verbal stimulation. Other sedative drugs or opioids were not used in this group. Intravenous paracetamol 1000 mg (15 mg/kg for patients under 50 kg) was given 30 min before the end of surgery. Anesthesia was considered inadequate if there was pain on pinching the surgical site using an artery forcep just prior to surgical incision. Patients with inadequate anesthesia were switched to GA according to protocol.

### Postoperative care and assessment

Two milli-grams of intravenous morphine sulphate was given every 5 min until the numerical rating scale (NRS) pain score was less than 4/10 in the PACU. In the general ward, oral analgesics were given once oral fluid was allowed. Regular oral paracetamol 500 mg was given every 6 h for 3 days or until discharge. Oral dihydrocodeine 30 mg as needed every 6 h was prescribed for 3 days or until discharge. Patients were told that they could request dihydrocodeine if their NRS pain score was over 3/10.

Oral fluid diet was allowed on postoperative day (POD) 0. The surgical team assessed for occurrences of postoperative surgical complications every morning and decided on patient suitability for discharge. Patients were assessed for possible complications related to the infraclavicular nerve block on POD 1, and they were reviewed again on POD 2 if complications could not be ruled out on the first assessment. Symptoms assessed included persistent paresthesia, tingling, abnormal sensation, and weakness. These assessments were made in the morning.

NRS pain scores (0–10), where 0 represented no pain, and 10 represented the worse possible pain, was assessed at rest and on movement of the operated upper limb (attempted wrist flexion). These were performed on arrival to the PACU, and at 1, 2, 24, and 48 h after surgery. Frequency and dose of rescue morphine used in the PACU and oral dihydrocodeine used in the ward were recorded daily in the morning. Occurrences of adverse effects including nausea, vomiting, pruritus and dizziness were also recorded every day. The Aonos four-point scale (AFPS) for postoperative emergence agitation was recorded 30 min after arrival at PACU. Patient satisfaction with anesthesia (0–10), where 0 was the least satisfaction, and 10 was the most satisfaction possible, was assessed on POD 1.

An investigator blinded to patient allocation conducted interviews at 3 and 6 months after surgery to collect the following information: NRS pain scores at rest and with movement (wrist flexion); patient satisfaction; health related quality of life measured using the Chinese version of SF12-v2 health survey [12]; psychological status as measured by the Hospital Anxiety and Depression Scale (HADS); and functional outcomes measured using the Disabilities of the Arm, Shoulder and Hand (QuickDASH) questionnaire and Patient Rated Wrist Evaluation (PRWE). Validated Chinese versions of QuickDASH and PRWE were used for Chinese patients [13, 14].

### Statistical analysis

The primary outcome was postoperative NRS pain score (0–10) with movement at 24 h after surgery. No suitable references for postoperative NRS pain scores at 24 h that could be used for sample size calculation was found. Based on a previous study on RA versus GA for hand surgery, the standard deviation estimate of the postoperative pain score from brachial plexus block was 2.86 [15]. To detect a difference in NRS pain score of 2.4/10 at a significance level of 0.05 and a power of 0.80, the minimum number of patients required per group was 23. A difference of 2.4 in NRS pain score was chosen because this has been shown to correspond to ‘much improvement’ in pain relief, which is clinically significant [16]. To take into account for possible dropouts, 26 patients were recruited into each group.

Secondary outcomes included postoperative NRS pain scores at other time points (apart from 24 h postop), postoperative analgesic consumption, patient satisfaction, adverse effects, upper limb functional scores (Patient-Rated Wrist Evaluation, QuickDASH), health related quality of life (SF12v2), and psychological status (HADS score). Patient demographic and operative data were analyzed using Student’s t-test and Chi-square test. Postoperative NRS pain scores between the two groups were expressed as median (interquartile range) and compared using the Mann-Whitney U test, as the distribution of the pain scores was non-normal. To adjust for multiple statistical comparisons, post-hoc Bonferroni correction was also applied for comparison of median postoperative NRS pain scores between the two groups for all observed time points. Pain scores between 0 and 48 h and between 3 and 6 months after surgery were also expressed as area under curve (AUC) weighted by the corresponding time interval. The weighted AUC is equivalent to a time weighted average of the pain scores for the specified time interval and is of the same scale as the NRS (0–10). The difference in weighted AUC between the GA and RA groups was compared by the Mann-Whitney U test. Analgesic consumption, functional outcomes (PRWE and QuickDASH), patient satisfaction, health related quality of life and psychological status were analyzed using the Student’s t-test. Incidence of adverse effects was compared using the Chi-square test or Fisher’s exact test as appropriate. The critical value for statistical significance was *P* = 0.05. Intention to treat analysis was done. The statistical software used was IBM SPSS Statistics for Windows, Version 25.0 (IBM Corp. USA).

## Results

**Fig. 1:**
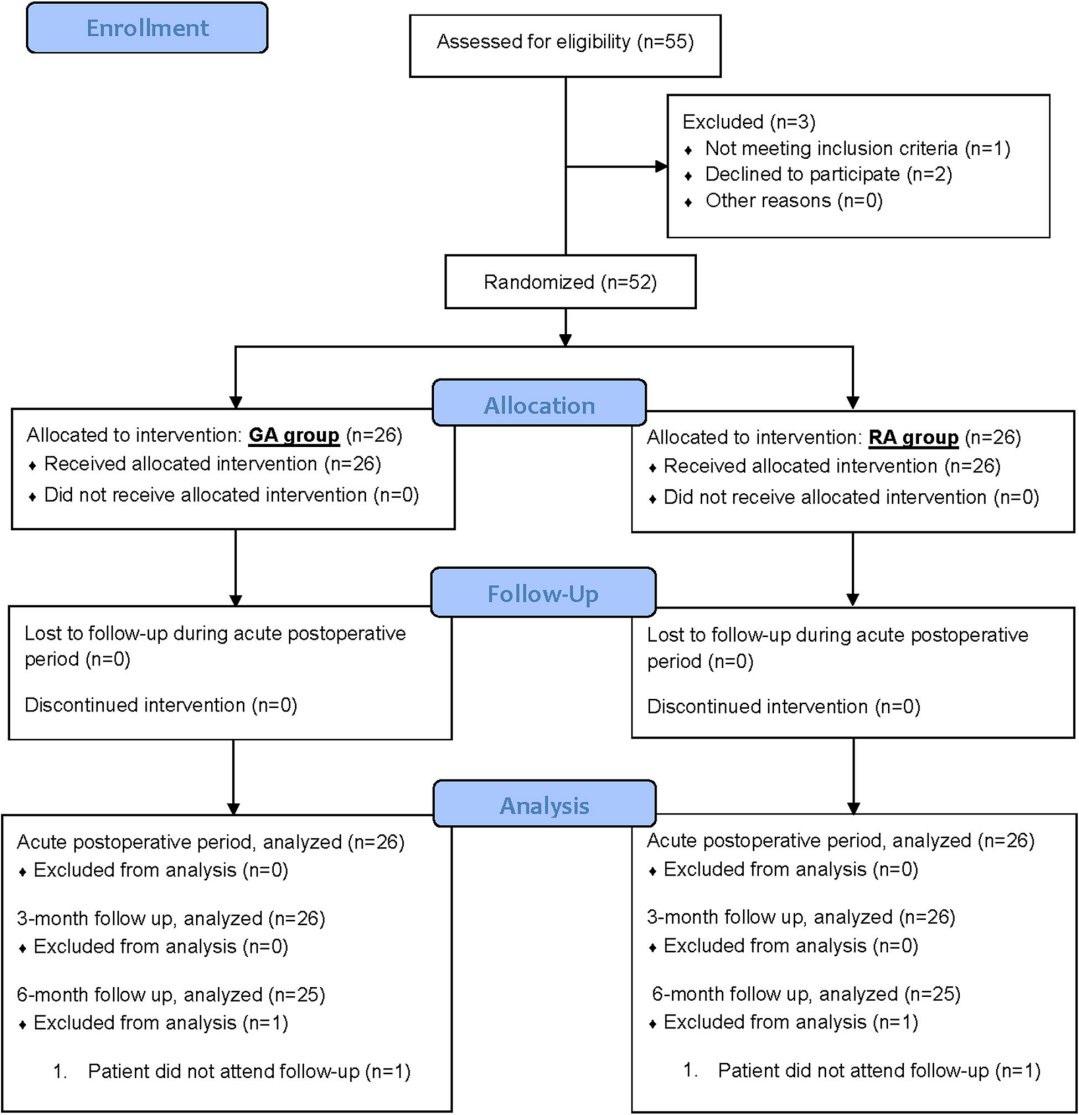
Flow diagram of patients enrolled in the study

Fifty-two patients in total (26 in each group) completed the study, which was conducted from May 2017 to January 2019. Fifty-five patients were assessed for eligibility, and 3 were excluded. Two patients declined to participate and 1 patient did not meet the inclusion criteria. All recruited patients completed the study for primary outcome measure and the results were used for analysis (Fig. 1).

Conversion to GA was required due to inadequate regional anesthetic block in 1 patient in the RA group. Intention to treat analysis was done. One patient from each group was lost to follow up at 6 months and were not included in the analysis for that time point. There were no significant differences between the two groups in patient demographics, duration of anesthesia, duration of surgery, and length of hospital stay (Table 1). Length of stay in the PACU was significantly longer in the GA group compared to the RA group (*p* = 0.023).

**Table 1:** Patient characteristics and operative data

Patient characteristics and operative data	GA (*n* = 26)	RA (n = 26)	*P* values
Sex (Female)	73.1% (19)	69.2% (18)	0.760
Age (year)	58.9 ± 12.8	59.2 ± 8.5	0.919
Body weight (kg)	60.1 ± 14.5	58.8 ± 9.5	0.518
ASA, I:II	30.8: 69.2% (8: 18)	53.8: 46.2% (14: 12)	0.092
Right: left distal radius fracture	42.3: 57.7% (11: 15)	42.3: 57.7% (11: 15)	1.000
Duration of anesthesia (min)	87.7 ± 21.8	97.6 ± 38.9	0.261
Duration of surgery (min)	56.1 ± 20.1	65.0 ± 37.3	0.288
Duration in PACU (min)	62.0 ± 23.7	48.8 ± 15.5	0.023^*^
Length of hospital stay (Days)	1.5 ± 0.81	1.5 ± 1.2	0.895

Values in mean ± SD or % (n)

Kg indicates kilograms; *ASA* American Society of Anesthesiologists physical status; *min* minutes; *PACU* post-anesthetic care unit; *SD* standard deviation

^*^ significantly different at *P* ≤ 0.05

**Fig. 2:**
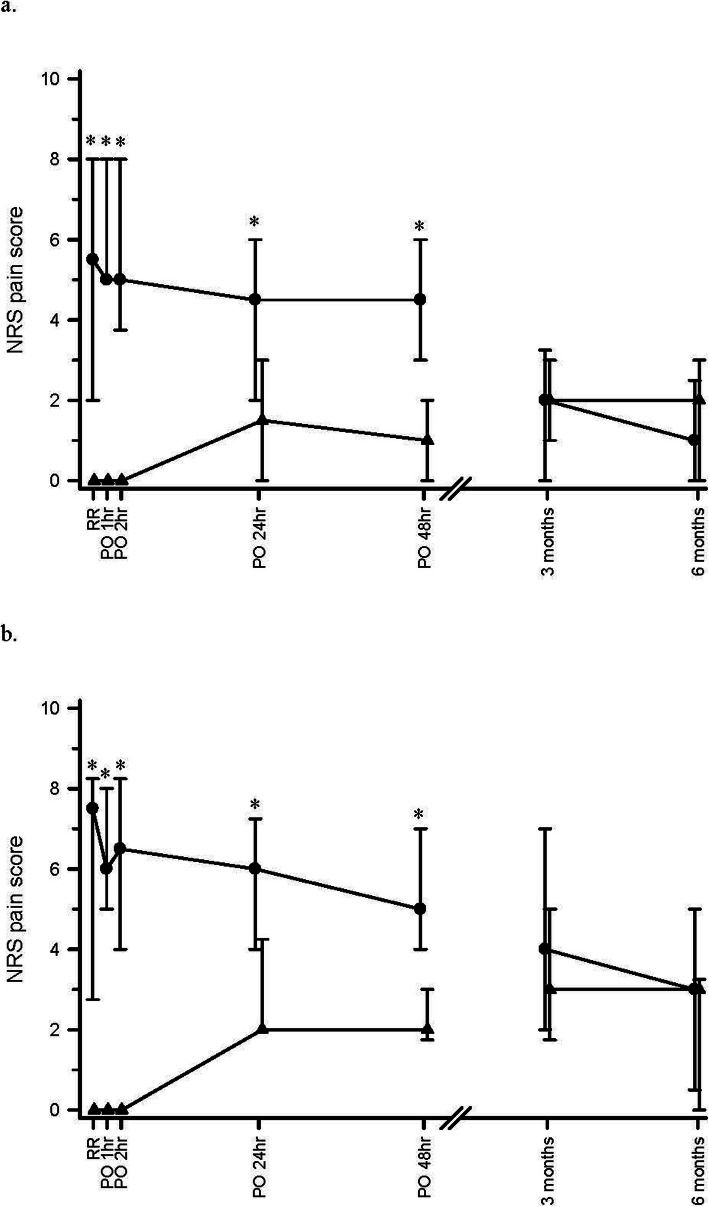
Postoperative numerical rating scale (NRS) pain scores of patients given regional anesthesia with infraclavicular nerve block (RA) or general anesthesia (GA) at rest (**a**) and during movement of the operated upper limb (**b**) at each recording time point. RR indicates on arrival to the post-anesthetic care unit; PO1hr = 1 h after surgery, PO2hr = 2 h after surgery; PO24hr = 24 h after surgery; PO48hr = 48 h after surgery; 3 and 6 months = 3 and 6 months after surgery. Solid circle represents GA; solid triangle represents RA. Values expressed in median [Interquartile range] * Significantly different at *P* ≤ 0.05. All differences in postoperative median NRS pain scores up to 48 h (at rest and with movement) remained statistically significant with adjusted *P* ≤ 0.05 even after post hoc adjustment with Bonferroni multiple comparisons

Postoperative median NRS pain scores at different time points are shown in Table 2 and Fig. 2. Patients in the RA group had significantly lower NRS pain scores than those in the GA group both at rest and during movement (attempted wrist flexion) on arrival to the PACU (*p*<0.001 for pain at rest and with movement), 1 h (*p*<0.001 at rest and with movement), 2 h (*p*<0.001 at rest and with movement), 24 h (*p* = 0.001 at rest and with movement) and 48 h (*p*<0.001 at rest and with movement) after surgery (Table 2). All of the above differences in NRS pain scores remained statistically significant with adjusted *p* < 0.05 after post-hoc application of Bonferroni multiple comparison correction. There were no significant differences between the two groups in NRS pain scores at rest and with movement (wrist flexion) at 3 and 6 months after surgery (Table 2). Postoperative NRS pain scores were also expressed as weighted AUC. The weighted AUC pain scores from 0 to 48 h after surgery was significantly lower in the RA group (*p* < 0.001 at rest and with movement). There were no differences in weighted AUC pain scores between the two groups from 3 to 6 months after surgery (Table 2).

**Table 2:** Postoperative NRS pain scores at rest and with movement

NRS pain scores	GA (*n* = 26)	RA (*n* = 26)	*P* values
*At rest*			
On arrival to PACU	5.5 [2–8]	0 [0–0]	< 0.001^*^
Postoperative 1 h	5 [5–8]	0 [0–0]	< 0.001^*^
Postoperative 2 h	5 [3.75–8]	0 [0–0]	< 0.001^*^
Postoperative 24 h	4.5 [2–6]	1.5 [0–3]	0.001^*^
Postoperative 48 h	4.5 [3–6]	1 [0–2]	< 0.001^*^
Postoperative 3 months	2 [0–3.25]	2 [1–3]	0.737
Postoperative 6 months	1 [0–2.5]	2 [0–3]	0.991
Weighted AUC 0–48 h	5.1 [2.8–5.8]	1.1 [0–2.1]	< 0.001*
Weighted AUC 3–6 months	1.5 [0.5–2.5]	1.5 [0.5–3.0]	0.992
*On movement of the operated limb*	(*n* = 26)	(*n* = 26)	
On arrival to PACU	7.5 [2.75–8.25]	0 [0–0]	< 0.001^*^
Postoperative 1 h	6 [5–8]	0 [0–0]	< 0.001^*^
Postoperative 2 h	6.5 [4–8.25]	0 [0–0]	< 0.001^*^
Postoperative 24 h	6 [4–7.25]	2 [2–4.25]	0.001^*^
Postoperative 48 h	5 [4–7]	2 [1.75–3]	< 0.001^*^
Postoperative 3 months	4 [2–7]	3 [1.75–5]	0.332
Postoperative 6 months	3 [0.5–5]	3 [0–3.25]	0.254
Weighted AUC 0–48 h	6.0 [4.5–6.9]	1.5 [1.4–3.2]	< 0.001*
Weighted AUC 3–6 months	3.5 [2.3–5.5]	3.3 [0.9–4.1]	0.254

Weighted AUC indicates weighted area under curve; *h* hours; *NRS* numerical rating scale

PACU indicates post-anesthetic care unit, values in median [Interquartile range]

Patient sample size at postoperative 6 months was 25 for both groups

^*^ Significantly different at *P* ≤ 0.05

Consumption of rescue morphine in the PACU was significantly higher in the GA group (*p* < 0.001) (Table 3). Oral paracetamol was given regularly to both groups of patients and there were no significant differences between the two groups (data not shown). There were no significant differences between groups in the consumption of rescue oral dihydrocodeine in the ward (Table 3).

**Table 3:** Postoperative analgesic consumption and incidence of postoperative adverse effects

Postoperative analgesic consumption	GA (*n* = 26)	RA (*n* = 26)	*P* values
*Morphine (mg)*			
Morphine consumption in the PACU	2.3 (0–3)	0 (0–0)	< 0.001^*^
*Oral Dihydrocodeine (mg) consumption*			
Postoperative 2 h	0 (0–0)	0 (0–0)	1.000
Postoperative 24 h	0 (0–0)	0 (0–0)	0.149
Postoperative 48 h	0 (0–0)	0 (0–0)	0.719
**Postoperative adverse effects**			
Emergence agitation (Aono’s four-point scale) in the PACU	1 (1–1)	1 (1–1)	0.057
Nausea	30.7% (13,49)	0.0% (NA)	0.004^*^
Vomiting	19.2% (4,34)	0.0% (NA)	0.050^*^
Dizziness	30.7% (13,49)	7.6% (−2,18)	0.075
Pruritus	0.0% (NA)	0.0% (NA)	1.000
Wound infection	0.0% (NA)	0.0% (NA)	1.000
Urinary retention	0.0% (NA)	3.8% (−4,12)	1.000
Others	11.5% (−1,25)	0.0% (NA)	0.235
*Symptoms of the operated upper limb*			
Paresthesia	23.1% (7,39)	11.5% (−1,25)	0.465
Tingling	23.1% (7,39)	7.7% (−2,18)	0.248
Abnormal sensation	19.2% (4,34)	3.8% (−4,12)	0.191
Weakness	30.8% (13,49)	7.7% (−2,18)	0.035^*^
Others	11.5% (−1,25)	0.0% (NA)	0.235

Values in median (IQR) or % (95% confidence interval)

PACU indicates post-anesthetic care unit; mg, milligram; *NA* not applicable

* Significantly different at *P* ≤ 0.05

There were no significant differences in the mean Aono’s score for emergence agitation in the PACU between the two groups (Table 3). Patients in the GA group were significantly more likely to experience nausea (*p* = 0.004) and vomiting (*p* = 0.050) after surgery (Table 3). There were no significant differences in the incidence of other adverse effects (Table 3). No immediate complications were reported. There were no significant differences in the incidence of paresthesia, tingling, and abnormal sensation over the operated limb (Table 3). Significantly more patients in the GA group experienced weakness in the acute postoperative period (*p* = 0.035) (Table 3). Patient satisfaction with anesthesia was significantly higher in the RA group on POD 1 (8.8 vs 7.3, *p* = 0.003) and at 3 months (8.4 vs 7.1, *p* = 0.007) after surgery, but there were no differences at 6 months. There were no differences in the proportion of patients willing to repeat the same anesthetic technique (data not shown).

There were no differences in functional scores as measured by PRWE and QuickDash (Disability/symptom score) at 3 and 6 months after surgery (Table 4). There were also no differences between the two groups in SF12-v2 and HADS at 3 and 6 months after surgery (data not shown).

**Table 4:** Functional scores: Patient Rated Wrist Evaluation (PRWE) and Disabilities of the arm, shoulder and hand questionnaire (QuickDASH)

Patient Rated Wrist Evaluation (PRWE)	GA (*n* = 26)	RA (*n* = 26)	*P* values
*Postoperative 3 months*			
Pain [0–50]	14 (8.3–17.8)	15 (7–22)	0.986
Specific Activities [0–60]	11.5 (3.3–20.5)	23.5 (3.8–31.3)	0.312
Usual Activities [0–40]	8 (2–20.3)	12 (2.5–22)	0.525
Total score [0–100]	23 (11.9–40.6)	35 (11.4–46.6)	0.618
*Postoperative 6 months*	(*n* = 25)	(*n* = 25)	
Pain [0–50]	10 (4–6)	10.5 (3–19)	0.657
Specific Activities [0–60]	6 (1.5–21)	9 (3–18.8)	0.820
Usual Activities [0–40]	8 (0–13.5)	4 (0–10.8)	0.213
Total score [0–100]	12 (7–34.8)	18.8 (4.1–34.4)	0.959
**Disabilities of the arm, shoulder and hand questionnaire (QuickDASH)**	(*n* = 26)	(*n* = 26)	
*Postoperative 3 months*			
Disability/symptom score [0–100]	21.6 (11.4–47.7)	34.1 (10.8–50)	0.839
*Postoperative 6 months*			
Disability/symptom score [0–100]	22.5 (11.4–32.3)	13.6 (9.1–24.4)	0.276

Values in median (IQR)

*PRWE Total score* Pain score + (Specific activities score + Usual activities score)/2

* Significantly different at *P* ≤ 0.05

## Discussion

RA with infraclavicular nerve block was associated with reduced NRS pain scores at rest and with movement compared to GA plus local anesthetic wound infiltration on arrival to the PACU, at 1, 2, 24 and 48 h after surgery. Patients in the RA group used significantly less morphine in the PACU, but oral dihydrocodeine consumption was similar between the two groups. There were no differences in pain scores, upper limb functional scores (PRWE and QuickDASH), psychological status (HADS) or health related quality of life (SF12v2) at 3 and 6 months after surgery.

Our results showed that RA was associated with better acute postoperative pain control compared to GA up to 48 h after distal radial fracture fixation. This is in contrast to the results from two previous randomized controlled trials conducted by Galos et al. and Rundgren et al. that compared RA versus GA for distal radial fracture fixation. In those 2 clinical trials, RA was not associated with overall analgesic benefit [7, 8]. Patients with RA had lower initial pain scores 2 h after surgery, but experienced rebound pain with worse pain scores 24 h after surgery [7, 8]. However, no significant rebound pain was observed in our study. A number of randomized controlled trials comparing RA versus GA for other types of upper limb surgery also did not demonstrate significant rebound pain [15, 17, 18]. All our patients were prescribed regular postoperative oral paracetamol which they took upon return to the ward before wearing off of the regional nerve block. Paracetamol taken regularly is recommended for postoperative pain control [19], and paracetamol is an effective analgesic medication [20, 21]. Since distal radial fracture surgery does not usually result in severe postoperative pain, regular oral paracetamol taken preventively was probably effective in controlling rebound pain. Unlike our study, standardized regular postoperative analgesic medication was not prescribed in the two clinical trials by Galos et al. and Rundgren et al. [7, 8]. In addition, our patients were also educated by ward staff about the possible experience of rebound pain and reinforced about the importance of taking regular analgesic medications, even in the absence of pain. Patient education was perhaps also useful in managing their expectations, which could affect their perception of pain. Our results suggest that regular preventive analgesic medications and patient education can attenuate rebound pain. This is in agreement with recommended strategies [11].

There are also other differences between our study and that of Galos et al. and Rundgren et al. that may have accounted for differences in our results. We examined both dynamic pain scores on movement of the upper limb and pain at rest, because we expected them to be distinctly different. In the clinical trials by Galos et al. and Rundgren et al., it is not clear whether pain scores assessed were at rest or with movement. The anesthetic technique was not standardized in the study by Galos et al. Differences in general anesthetic technique such as the choice of inhalational versus propofol total intravenous anesthesia can affect postoperative pain scores and opioid consumption [22–24]. Finally, 7 of the 44 patients had an inadequate block in the study by Rundgren et al., versus only 1 out of 26 patients in our study.

In this study, single shot infraclavicular nerve block was associated with improved postoperative analgesia for 48 h, which is beyond the pharmacological duration of the local anesthetic. This suggests that infraclavicular nerve block had preventive analgesic effects. Preventive analgesia is defined as a reduction in postoperative pain and/or analgesic consumption that persists longer than the clinical duration of the target drug (5.5 half-lives) [25]. We hypothesize that infraclavicular nerve block prevented central sensitization by inhibiting nociceptive input into the spinal cord, thereby producing preventive analgesia and prolonged analgesia [3, 26]. There have been some other clinical studies that also showed improved analgesia up to 48 h after surgery with single shot regional nerve blocks [27, 28].

We evaluated the opioid sparing effects of RA versus GA. Morphine consumption in the PACU was significantly lower in the RA group compared to the GA group, which is similar to other studies comparing RA versus GA for upper limb surgery [7, 15, 18]. However, no significant difference in oral dihydrocodeine consumption in the ward was observed. The absence of difference in rescue oral analgesic consumption may be because the pain scores after surgery were not very high. The median NRS pain scores at rest in the GA group were 4.5/10 at 24 and 48 h after surgery, which corresponds to pain of moderate intensity [29]. It was up to the patient whether or not to request oral dihydrocodeine. Although patients in the GA group experienced more pain, it may not have been severe enough to cause a significant difference in oral analgesic request. Patient satisfaction with anesthesia after surgery was significantly higher in the RA group on POD 1. This is probably due to better acute postoperative pain control and reduced nausea and vomiting in patients given RA. It has been shown that patient dissatisfaction is strongly associated with worse pain control as well as nausea and vomiting [30, 31]. Interestingly, patients in the RA group also had higher levels of satisfaction at 3 months after surgery, even though there was no difference in pain scores at that time point. Perhaps a trend towards better functional scores (PRWE and QuickDash) in the RA group contributed to higher levels of satisfaction. However, these differences were not statistically significant, and was probably not the only reason for this observation. In addition, although RA group patients had higher levels of satisfaction, the proportion of patients who would repeat the same anesthetic technique was similar between the two groups. Both GA and RA patients had good satisfaction scores (8.8 vs 7.3), and over 80% of both groups of patients would repeat the same anesthetic technique. The difference in patient satisfaction was probably not large enough to significantly affect this outcome. Another reason could be the concern of potential nerve block related complications.

We found no differences in longer-term outcomes between the 2 groups. This is in agreement with Galos et al. and Rundgren et al., which found no differences in pain, DASH, short musculoskeletal function assessment, PRWE, and EuroQol-5 Dimensions-3 Levels [7, 8]. On the other hand, an observational study involving 187 patients showed that RA was associated with reduced pain scores and higher upper limb functional scores (DASH) at 3 and 6 months after distal radial fracture fixation [32]. The relationship between RA and longer-term outcomes for distal radial fracture fixation remains unclear.

There were some limitations in this study. The patients were not blinded, and this can introduce bias. However, it was not possible to blind the patients in this study. The investigators performing patient assessment and data collection were blinded, and bias does not appear to be a significant issue when the observer is blinded [33]. Another limitation was that our study was not powered to look for differences in long-term pain score and functional outcomes at 3 and 6 months. The main focus of this study was to compare RA versus GA for acute postoperative pain control, but we recognize that long-term outcomes are also important. An additional limitation was that there may have been some variation in the amount of medication given to the two groups of patients intraoperatively, because the study protocol allowed the attending anesthetist to give medications within a given dose range. Airway management was also not standardized (laryngeal mask airway or endotracheal intubation was allowed). In addition, there was also some variation in the volume of local anesthetic used for the infraclavicular nerve block (between 15 and 20 ml), which could affect the quality of the block. However, the vast majority of the infraclavicular nerve blocks were sufficient for surgical anesthesia, indicating good block quality. There was 1 failed block in this study. This may have been reduced if a single anesthesiologist with special expertise in RA performed all the ultrasound guided infraclavicular nerve blocks. However, this was not done in order to make the results more generalizable to everyday clinical practice.

## Conclusion

Our study suggests that RA improves acute postoperative pain control compared to GA for distal radial fracture fixation. RA was also associated with higher patient satisfaction, and less nausea and vomiting. Preventive regular analgesic medication in combination with RA is probably important to improve postoperative pain control and prevent rebound pain.

## Data Availability

All data generated or analyzed during this study are presented in this manuscript. The additional datasets are also available from the corresponding author on reasonable request.

## Abbreviations

AFPS: Aonos four point scale
ALT: Alanine aminotransferase
ASA: American Society of Anesthesiologist
AST: Aspartate aminotransferase
AUC: Area under curve
ECG: Electrocardiogram
GA: General anesthesia
HADS: Hospital Anxiety and Depression Scale
INR: International normalized ratio
MAC: Minimum alveolar concentration
NRS: Numerical rating scale
NSAIDs: Non-steroidal anti-inflammatory drugs
PACU: Post-anesthetic care unit
PO1hr: 1 hour after surgery
PO2hr: 2 hours after surgery
PO24hr: 24 hours after surgery
PO48hr: 48 hours after surgery
POD: Postoperative day
PRWE: Patient Rated Wrist Evaluation
QuickDASH: Quick Disabilities of the Arm, Shoulder and Hand
RA: Regional anesthesia
RR: On arrival to the post-anesthetic care unit
SF12v2: Short Form 12 item (version 2) health survey
